# A Practicable Elastoplastic Damage Model for Concrete with Decoupled Plasticity and Damage

**DOI:** 10.3390/ma19204334

**Published:** 2026-10-15

**Authors:** Yupeng Zhang, Hu Qi, YongRong You, Dechun Lu

**Affiliations:** 1 Key Laboratory of Urban Security and Disaster Engineering of the Ministry of Education, Beijing University of Technology, Beijing 100124, China; 2 School of Earthquake Engineering and Building Safety, University of Emergency Management, Langfang 065201, China; 3 Key Laboratory of Building Collapse Mechanism and Disaster Prevention, China Earthquake Administration, Sanhe 065201, China; 4 School of Architecture and Civil Engineering, Xiamen University, Xiamen 361005, China

**Keywords:** concrete, elastic–plastic damage model, damage mechanics, multiaxial loading, finite element analysis

## Abstract

In this paper, a practicable elastoplastic damage model with decoupled plasticity and damage for concrete is proposed. Plastic strain is evaluated in the effective stress space through an empirical relation, allowing the plastic and damage processes to be treated separately and simplifying the constitutive calculation. Within an irreversible thermodynamics framework, the damage criterion is formulated in terms of the damage energy release rate derived from a modified elastic Helmholtz free energy expression. The free energy function is separated into hydrostatic and deviatoric parts. The direct hydrostatic contribution to the damage-driving force is omitted, while a stress-state-dependent coefficient modifies the deviatoric contribution under multiaxial stress states. Spectral decomposition of the effective stress tensor provides positive and negative parts, enabling separate evolution of tensile and compressive damage. Independent tensile and compressive damage variables are combined through a weighted scalar damage measure to represent their interaction and the tension–compression softening effect. The proposed model retains a simple mathematical structure while incorporating plastic deformation, stiffness degradation, damage evolution, and loading-state dependence within a unified framework. Comparison with experimental data shows that the relative error and peak stress error for the biaxial strength envelope are 1.5% and 0.9%, respectively, while the peak stress errors for uniaxial and biaxial compression are 0.71% and 0.55%, respectively. The first three natural circular frequencies of the Koyna concrete dam predicted by the model are 18.86, 49.98, and 68.16 rad/s, showing close agreement with published reference results. These results demonstrate the applicability of the proposed formulation to nonlinear numerical analysis of concrete materials and structures.

## 1. Introduction

Reliable structural finite-element prediction requires an adequate description of concrete nonlinearity. Concrete contains numerous microcracks and microvoids, whose initiation, propagation, and coalescence lead to stiffness degradation, strain softening, strength deterioration, and, in some cases, strain localization. These nonlinear features become more evident under monotonic, cyclic, dynamic, and multiaxial loading conditions, and they directly influence the reliability of structural response prediction. Damage mechanics provides an effective framework for describing microcrack-induced stiffness deterioration and post-peak degradation, while recent damage-based models have further improved the simulation of strain localization and mesh-size sensitivity in finite element analysis [1].

However, a purely damage-based model is generally insufficient to describe irreversible deformation, plastic energy dissipation, and volumetric deformation. Plasticity-based models can represent yielding, hardening–softening behavior, and permanent strain, but classical plasticity alone cannot adequately capture unloading stiffness degradation caused by damage evolution. Therefore, a reasonable concrete constitutive model should combine damage mechanics and plasticity theory. Coupled damage–plasticity models can simultaneously account for stiffness degradation and irreversible deformation, and have become an important approach for describing the nonlinear response of concrete up to failure [2,3,4].

In recent years, damage–plasticity models have been further developed for different loading conditions and material behaviors. For multiaxial stress states, confinement-enhanced and anisotropic damage–plasticity formulations have been developed to capture confinement effects, tension–compression asymmetry, stiffness recovery after crack closure, and direction-dependent damage evolution [2,3,4,5]. In particular, enhanced CDP-based formulations have been developed for monotonic and cyclic triaxial compression by modifying the failure surface, introducing hydrostatic-pressure-dependent parameters, and establishing confinement-dependent hardening–softening and damage evolution rules [5]. For cyclic loading, the description of unloading–reloading paths, crack closure and reopening, hysteretic energy dissipation, and damage accumulation is especially important. Related studies have improved the damage evolution law and cyclic response description of concrete, showing that the coupling between damage degradation and plastic deformation is necessary for reproducing cyclic stiffness degradation and residual deformation [6,7,8].

Concrete damage plasticity is widely implemented in commercial finite-element programs to represent tensile cracking, compressive crushing, stiffness loss, and irreversible deformation. Recent work has examined the calibration and optimization of CDP parameters, including dilation angle, viscosity parameter, tensile and compressive damage variables, stress–strain relationships, and mesh sensitivity. These studies have broadened the use of CDP-based models for reinforced concrete structures, cracked members, and ultra-high-performance concrete under monotonic and cyclic loading conditions [8,9,10].

For concrete under complex actions, the constitutive description may also need to account for rate effects, fatigue, and environmental degradation. Thermoelastic–plastic damage and dynamic damage formulations have been proposed to capture strain-rate effects, thermal softening, damage evolution, and dynamic strength enhancement under impact or seismic loading conditions [11,12]. Equivalent fatigue formulations have also been incorporated into the CDP framework for high-cycle fatigue analysis, while freeze–thaw models based on modified stress–strain relations and damage factors have been used to represent deterioration of the mechanical response caused by environmental exposure [13,14].

Although these studies have significantly improved the description of concrete nonlinear behavior, many existing damage–plasticity formulations involve complex coupling between plasticity and damage, multiple material parameters, and iterative constitutive procedures, which may complicate parameter identification and numerical implementation in structural analyses. In addition, the influence of the multiaxial stress state on damage evolution is not always represented explicitly in relatively simple isotropic damage formulations. Therefore, there remains a need for an elastic–plastic damage formulation that can describe stiffness degradation, irreversible deformation, tensile–compressive damage evolution, and multiaxial stress-state effects within a comparatively simple constitutive framework.

Accordingly, the objective of this study is to develop an elastic–plastic damage model for concrete based on the effective stress space and damage energy release rate. The main features of the proposed formulation are: (i) plastic deformation and damage evolution are treated separately in the effective stress space to simplify their coupling; (ii) tensile and compressive damage are described by separate scalar damage variables and combined through a weighted damage parameter; and (iii) a stress-state-dependent coefficient γ is introduced to modify the deviatoric damage-driving contribution under multiaxial loading. The model is subsequently evaluated against experimental results under uniaxial, biaxial, and cyclic loading conditions and applied to the nonlinear analysis of the Koyna concrete dam.

## 2. Theoretical Basis

### 2.1. Elastic-Plastic Damage Constitutive Relation

The formulation combines effective stress-space plasticity with isotropic continuum damage mechanics and is developed for small-strain, isothermal, and rate-independent conditions. Under the strain equivalence hypothesis, the total strain of the damaged configuration is taken to be identical to that of the associated effective configuration. Both are additively decomposed into elastic and plastic parts [15,16,17]:

(1) $$\left\{\begin{array}{c} \varepsilon_{ij}={\varepsilon_{ij}}^{e}+{\varepsilon_{ij}}^{p}\\ {\bar{\varepsilon }}_{ij}={\bar{\varepsilon }}_{ij}^{e}+{{\bar{\varepsilon }}_{ij}}^{p} \end{array}\right.\Rightarrow \varepsilon_{ij}=\varepsilon_{ij}$$

Here, $\varepsilon_{ij}$ denotes the total strain tensor, decomposed into the elastic strain tensor ${\varepsilon_{ij}}^{e}$ and the plastic strain tensor ${\varepsilon_{ij}}^{p}$, ${\bar{\varepsilon }}_{ij}$ denotes the effective total strain tensor, ${{\bar{\varepsilon }}_{ij}}^{e}$ denotes the effective elastic strain tensor,${{\bar{\varepsilon }}_{ij}}^{p}$ denotes the effective plastic strain tensor, and ${\bar{E}}_{ijkl}$ is the effective (undamaged) constitutive operator.

Differentiating Equation (1) with respect to time gives:

(2) $$\left\{\begin{array}{c} {\dot{\varepsilon }}_{ij}={{\dot{\varepsilon }}_{ij}}^{e}+{{\dot{\varepsilon }}_{ij}}^{p}\\ {\dot{\bar{\varepsilon }}}_{ij}={\dot{\bar{\varepsilon }}}_{ij}^{e}+{\dot{\bar{\varepsilon }}}_{ij}^{p} \end{array}\right.\Rightarrow {\dot{\varepsilon }}_{ij}={\dot{\bar{\varepsilon }}}_{ij}$$

Using strain equivalence, the stress tensor in the effective (undamaged) configuration is written as:

(3) $${\bar{\sigma }}_{ij}={\bar{E}}_{ijkl}{\varepsilon_{ij}}^{e}={\bar{E}}_{ijkl}(\varepsilon_{ij}-{\varepsilon_{ij}}^{p})$$

Combining the strain equivalence hypothesis with Hooke’s law gives the effective stress rate:

(4) $${\dot{\bar{\sigma }}}_{ij}={\bar{E}}_{ijkl}{{\dot{\varepsilon }}_{ij}}^{e}={\bar{E}}_{ijkl}({\dot{\varepsilon }}_{ij}-{{\dot{\varepsilon }}_{ij}}^{p})$$

The damage configuration counterpart of Equation (3) is given as follows:

(5) $$\sigma_{ij}=E_{ijkl}(d){\varepsilon_{ij}}^{e}=E_{ijkl}(d)(\varepsilon_{ij}-{\varepsilon_{ij}}^{p})$$

Here, denotes the fourth-order elasticity tensor for the damaged configuration.

A weighted scalar damage variable is adopted. Following the effective stress concept of Kachanov [18], the Cauchy stress tensor $\sigma_{ij}$ is related to its effective counterpart ${\bar{\sigma }}_{ij}$ by:

(6) $$\sigma_{ij}=(1-d){\bar{\sigma }}_{ij}$$

where *d* is the weighted scalar damage variable, with 0 < *d* < 1, where *d* = 0 denotes the undamaged state and *d* = 1 denotes complete loss of stiffness.

Substitution of Equations (3) and (5) into Equation (6) gives:

(7) $$E_{ijkl}=(1-d){\bar{E}}_{ijkl}$$

(8) $$\sigma_{ij}=(1-d){\bar{E}}_{ijkl}(\varepsilon_{ij}-{\varepsilon_{ij}}^{p})$$

Differentiating Equation (8) with respect to time yields the constitutive rate equation of the present elastic–plastic damage model:

(9) $${\dot{\sigma }}_{ij}=(1-d){\bar{E}}_{ijkl}{\dot{\varepsilon }}_{ij}^{e}-\dot{d}{\bar{E}}_{ijkl}\varepsilon_{ij}^{e}$$

**Figure 1:**
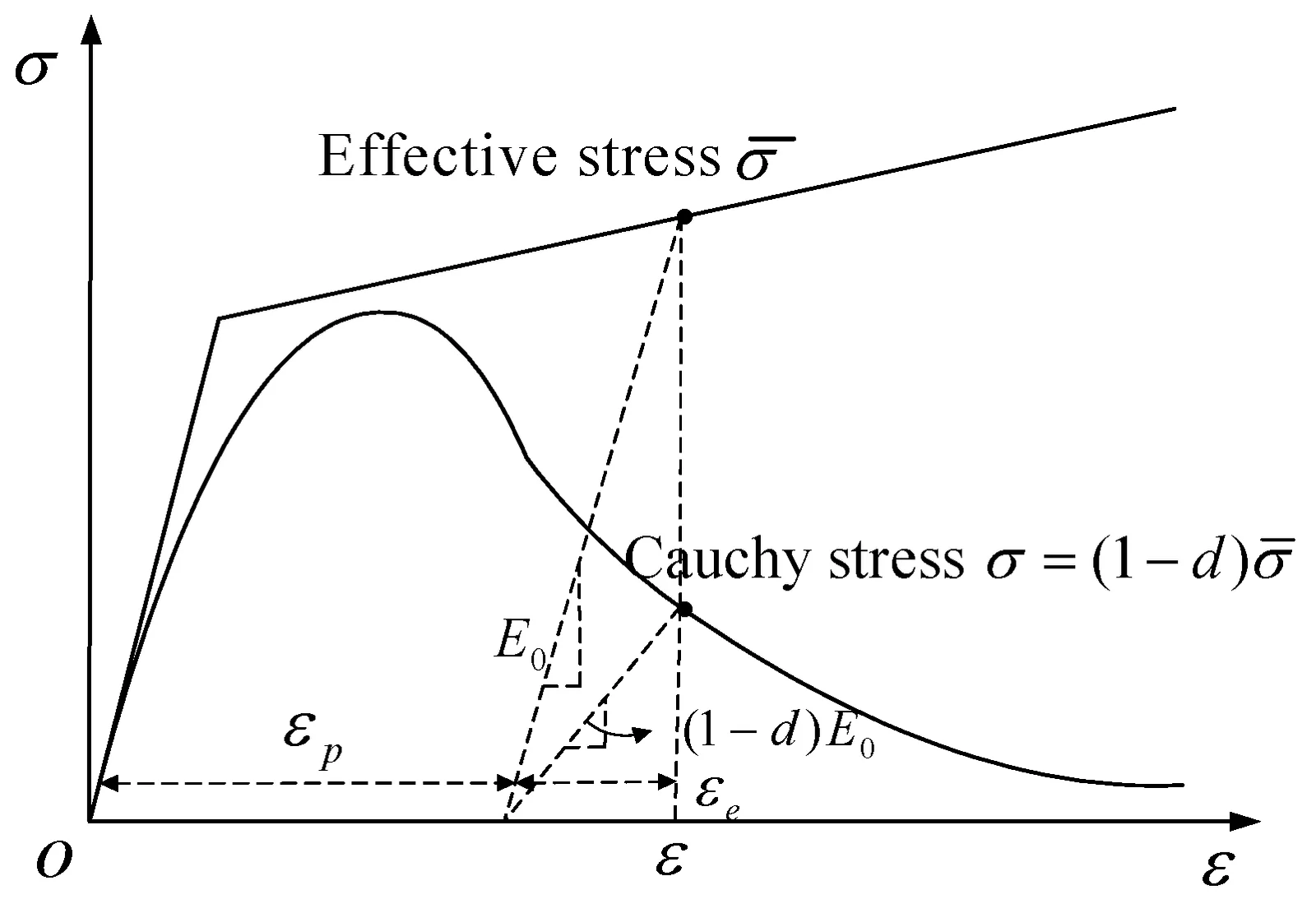
The model under 1D loading.

Equation (9) shows that the constitutive update requires determination of the plastic strain tensor and the damage variable. The corresponding uniaxial response is illustrated in Figure 1.

### 2.2. Framework of Thermodynamics

Under small-strain, isothermal, and rate-independent conditions, the Helmholtz free energy (HFE) ψ is taken as a function of the following internal variables:

(10) $$\psi (\varepsilon_{ij}^{e},\kappa ,d)=\psi^{e}(\varepsilon_{ij}^{e},d)+\psi^{p}(\kappa ,d)$$

where κ is the plastic hardening variable; $\psi^{e}(\varepsilon_{ij}^{e},d)$ is the elastic HFE; $\psi^{p}(\kappa ,d)$ is the plastic Helmholtz free energy.

The second law of thermodynamics gives the Clausius–Duhem inequality for an irreversible, not necessarily adiabatic, process as:

(11) $$\dot{\eta }=\sigma_{ij}{\dot{\varepsilon }}_{ij}-\dot{\psi }\geq 0$$

Here, η denotes entropy.

Differentiating Equation (10) with respect to time and inserting the result into Equation (11) yields:

(12) $$\left[\sigma_{ij}-\frac{\partial \psi^{e}}{\partial \varepsilon_{ij}^{e}}\right]{\dot{\varepsilon }}_{ij}^{e}-\frac{\partial \psi }{\partial d}\dot{d}+\left[\sigma_{ij}{\dot{\varepsilon }}_{ij}^{p}-\frac{\partial \psi^{p}}{\partial \kappa }\dot{\kappa }\right]\geq 0$$

Because this inequality must hold for every admissible set of internal variables, the following relations are obtained:

(13) $$\sigma_{ij}=\frac{\partial \psi^{e}}{\partial \varepsilon_{ij}^{e}}$$

(14) $$-\frac{\partial \psi }{\partial d}\dot{d}\geq 0\Rightarrow Y\dot{d}\geq 0$$

(15) $$\sigma_{ij}{\dot{\varepsilon }}_{ij}^{p}-\frac{\partial \psi^{p}}{\partial \kappa }\dot{\kappa }\geq 0$$

where Y is a thermodynamic force conjugate to the damage variable, referred to as the damage energy release rate (DERR).

Equation (13) relates Cauchy stress to strain. Equation (14) gives the damage dissipation inequality, and $-\frac{\partial \psi }{\partial d}\dot{d}$ represents the energy released during damage evolution.

The damage energy release rate Y, thermodynamically conjugate to the damage variable d, is written as:

(16) $$Y=-\frac{\partial \psi }{\partial d}$$

If damage is assumed to modify only the elastic part of ψ, while the plastic part remains unaffected, Equation (16) reduces to:

(17) $$Y=Y^{e}=-\frac{\partial \psi^{e}}{\partial d}$$

Substituting Equations (16) and (17) into Equation (14), one obtains:

(18) $$-Y\dot{d}\geq 0$$

Equation (18) imposes the non-negative damage dissipation condition $Y\dot{d}\geq 0$, which is analogous to the Drucker stability requirement in plasticity. It ensures that the damage process does not generate energy and that damage evolution is thermodynamically admissible.

(19) $$g=g(\varepsilon_{ij},Y,d)=0$$

The loading evolution of the damage variable is then written as:

(20) $$\dot{d}={\dot{\lambda }}_{d}\frac{\partial g}{\partial Y}$$

where ${\dot{\lambda }}_{d}$ is the damage consistency parameter.

## 3. The Elastic Plastic Damage Model of This Paper

Before presenting the constitutive formulation, the following basic assumptions are adopted. The model is formulated under small-strain, isothermal, and rate-independent conditions. The strain equivalence hypothesis is assumed between the damaged and effective configurations. Plastic deformation is evaluated in the effective stress space, while material degradation is described within the framework of isotropic continuum damage mechanics. Damage is assumed to affect the elastic properties of the material, whereas its influence on the plastic free energy component is neglected.

### 3.1. Plastic Deformation

The plastic strain is evaluated in the effective stress space. To avoid the relatively complex numerical procedures associated with classical plasticity, the simplified evolution law proposed by Faria et al. [19] is adopted:

(21) $${{\dot{\varepsilon }}_{ij}}^{p}=\beta_{p}EH({\dot{d}}^{-})\langle I_{{\bar{\sigma }}_{ij}}{\dot{\varepsilon }}_{ij}\rangle {\bar{D}}_{ijkl}I_{{\bar{\sigma }}_{ij}}$$

where $\beta_{p}$ is a material parameter controlling the magnitude of plastic strain evolution, E is the elastic modulus, and ${\bar{D}}_{ijkl}$ is the effective flexibility tensor. $H\left({\overset{\cdot }{d}}^{-}\right)$ denotes the Heaviside function associated with the compressive damage rate ${\overset{\cdot }{d}}^{-}$, which activates the plastic strain evolution when compressive damage develops.

This simplified kinematic treatment avoids several operations required in classical plasticity. Because the model is intended for long-duration seismic simulations, a compact constitutive update is preferred [19].

The effective stress increment is written as:

(22) $${\dot{\bar{\sigma }}}_{ij}={\bar{E}}_{ijkl}({\dot{\varepsilon }}_{ij}-{{\dot{\varepsilon }}_{ij}}^{p})$$

Substituting Equation (21) into Equation (22) gives:

(23) $${\dot{\bar{\sigma }}}_{ij}={\bar{E}}_{ijkl}({\dot{\varepsilon }}_{ij}-\beta_{p}EH({\dot{d}}^{-})\langle I_{{\bar{\sigma }}_{ij}}\dot{\varepsilon }\rangle {\bar{D}}_{ijkl}I_{{\bar{\sigma }}_{ij}})$$

### 3.2. Damage Variables

#### 3.2.1. The Helmholtz Free Energy (HFE) in Tao and Phillips [20]

To determine the damage variable, the expression for the Helmholtz free energy (HFE) should first be specified. The HFE adopted in the model of Tao and Phillips [20] is modified in the present study.

Using the effective stress and strain obtained from Section 2.1, the undamaged HFE is written as:

(24) $$\bar{\psi^{e}}=\frac{1}{2}{\bar{\sigma }}_{ij}{\bar{D}}_{ijkl}{\bar{\sigma }}_{kl}=\frac{1}{2}{\bar{\sigma }}_{ij}\varepsilon_{ij}^{e}$$

The damaged elastic HFE is obtained by incorporating stiffness degradation through the strain equivalence assumption:

(25) $$\psi^{e}=(1-d)\bar{\psi^{e}}=\frac{1}{2}\sigma_{ij}D_{ijkl}\sigma_{kl}=\frac{1}{2}(1-d){\bar{\sigma }}_{ij}{\bar{D}}_{ijkl}{\bar{\sigma }}_{kl}=\frac{1}{2}\sigma_{ij}\varepsilon_{ij}^{e}$$

The effective stress tensor ${\bar{\sigma }}_{ij}$ is then additively separated into deviatoric ${\bar{s}}_{ij}$ and hydrostatic parts ${\bar{\sigma }}_{\mathrm{mm}}/3$:

(26) $${\bar{\sigma }}_{ij}={\bar{s}}_{ij}+\frac{1}{3}{\bar{\sigma }}_{mm}\delta_{ij}$$

Here, the hydrostatic component represents the mean effective stress, whereas the deviatoric component describes the stress state after removing the mean stress and satisfies ${\bar{S}}_{kk}$. Substituting this decomposition into Equation (25) leads to Equation (27).

Substitution of the decomposition in Equation (26) into Equation (25) gives:

(27) $$\psi^{e}=\frac{1}{2}(1-d)({\bar{s}}_{ij}{\bar{D}}_{ijkl}{\bar{s}}_{kl}+\frac{1}{3}{\bar{\sigma }}_{mm}{\bar{s}}_{ij}{\bar{D}}_{ijkl}\delta_{kl}+\frac{1}{3}{\bar{\sigma }}_{mm}\delta_{ij}{\bar{D}}_{ijkl}{\bar{s}}_{kl})+\frac{1}{2}(1-d)(\frac{1}{9}{\left({\bar{\sigma }}_{mm}\right)}^{2}\delta_{ij}{\bar{D}}_{ijkl}\delta_{kl})$$

where ${\hat{\bar{s}}}_{ij}$ is the deviatoric part of the effective principal stress, and is the hydrostatic part of the effective principal stress.

Using Voigt markers, the effective concrete flexibility tensor can be expressed as:

(28) $${\bar{D}}_{ijkl}=\left[\begin{array}{cccccc} a & b & b & & & \\ b & a & b & & & \\ b & b & a & & & \\ & & & c & & \\ & & & & c & \\ & & & & & c \end{array}\right]$$

where v is Poisson’s ratio, and E is the initial elastic modulus, corresponding to $E_{0}$, as shown in Figure 1.

(29) $$\frac{1}{3}{\bar{\sigma }}_{mm}{\bar{s}}_{ij}{\bar{D}}_{ijkl}\delta_{kl}=\frac{1}{3}{\bar{\sigma }}_{mm}\delta_{ij}{\bar{D}}_{ijkl}{\bar{s}}_{kl}=\frac{1}{3}{\bar{\sigma }}_{mm}(a+2b)({\bar{s}}_{11}+{\bar{s}}_{22}+{\bar{s}}_{33})$$

(30) $${\bar{s}}_{11}+{\bar{s}}_{22}+{\bar{s}}_{33}={\bar{\sigma }}_{11}+{\bar{\sigma }}_{22}+{\bar{\sigma }}_{33}-{\bar{\sigma }}_{mm}=0$$

(31) $$\frac{1}{3}{\bar{\sigma }}_{mm}{\bar{s}}_{ij}{\bar{D}}_{ijkl}\delta_{kl}=\frac{1}{3}{\bar{\sigma }}_{mm}\delta_{ij}{\bar{D}}_{ijkl}{\bar{s}}_{kl}=0$$

(32) $$\psi^{e}=\frac{1}{2}(1-d)({\bar{s}}_{ij}{\bar{D}}_{ijkl}{\bar{s}}_{kl})+\frac{1}{2}(1-d)(\frac{1}{9}{\left({\bar{\sigma }}_{mm}\right)}^{2}\delta_{ij}{\bar{D}}_{ijkl}\delta_{kl})$$

Thus, the first term of Equation (32) only includes the principal deviatoric stress, which can be considered the deviatoric component of the HFE, and the second term is the hydrostatic component.

Because the hydrostatic strain contribution to concrete damage is smaller than the deviatoric contribution, Tao and Phillips [20] introduced the damage multiplier (damage reduction factor) β to represent this reduction:

(33) $$\psi^{e}=\frac{1}{2}(1-d)({\bar{s}}_{ij}{\bar{D}}_{ijkl}{\bar{s}}_{kl})+\frac{1}{2}(1-\beta d)(\frac{1}{9}{\left({\bar{\sigma }}_{mm}\right)}^{2}\delta_{ij}{\bar{D}}_{ijkl}\delta_{kl})$$

β is restricted to the interval 0 ≤ β ≤ 1. Tao and Phillips [20] expressed the damage reduction factor β as follows:

(34) $$\beta =1-\frac{1}{1+cYe^{\left(-dY\right)}}$$

Here, *Y* denotes the damage energy release rate (DERR), while c and d are parameters of the damage reduction function.

Substituting Equation (33) into Equation (17), one can obtain the following:

(35) $$Y=-\frac{\partial \psi^{e}}{\partial d}=\frac{1}{2}({\bar{s}}_{ij}{\bar{D}}_{ijkl}{\bar{s}}_{kl})+\frac{1}{2}\beta (Y)(\frac{1}{9}{\left({\bar{\sigma }}_{mm}\right)}^{2}\delta_{ij}{\bar{D}}_{ijkl}\delta_{kl})$$

#### 3.2.2. Spectral Decomposition of the Effective Stress Tensor

To distinguish tension- and compression-driven damage, the effective stress tensor $\bar{\sigma }$ is spectrally split into positive and negative parts (${{\bar{\sigma }}_{ij}}^{+}$, ${{\bar{\sigma }}_{ij}}^{-}$) [16,19,21,22,23,24]:

(36) $${\bar{\sigma }}_{ij}={{\bar{\sigma }}_{ij}}^{+}+{{\bar{\sigma }}_{ij}}^{-}$$

(37) $${\bar{\sigma }}_{ij}=\sum_{k=1}^{3}{\hat{\bar{\sigma }}}^{\left(k\right)}P_{i}^{\left(k\right)}P_{j}^{\left(k\right)}$$

(38) $${{\bar{\sigma }}_{ij}}^{+}=H({\hat{\bar{\sigma }}}^{\left(1\right)}){\hat{\bar{\sigma }}}^{\left(1\right)}P_{i}^{\left(1\right)}P_{j}^{\left(1\right)}+H({\hat{\bar{\sigma }}}^{\left(2\right)}){\hat{\bar{\sigma }}}^{\left(2\right)}P_{i}^{\left(2\right)}P_{j}^{\left(2\right)}+H({\hat{\bar{\sigma }}}^{\left(3\right)}){\hat{\bar{\sigma }}}^{\left(3\right)}P_{i}^{\left(3\right)}P_{j}^{\left(3\right)}$$

Effective principal stress ${\hat{\bar{\sigma }}}_{ij}^{k}$ can be calculated as follows:

(39) $${\hat{\bar{\sigma }}}_{ij}^{\left(k\right)}=P_{i}^{\left(k\right)}{\bar{\sigma }}_{ij}P_{j}^{\left(k\right)}$$

Substituting Equation (39) into Equation (38), one can obtain the following:

(40) $${{\bar{\sigma }}_{ij}}^{+}=H({\hat{\bar{\sigma }}}^{\left(1\right)})P_{i}^{\left(1\right)}{\bar{\sigma }}_{ij}P_{j}^{\left(1\right)}P_{i}^{\left(1\right)}P_{j}^{\left(1\right)}+H({\hat{\bar{\sigma }}}^{\left(2\right)})P_{i}^{\left(2\right)}{\bar{\sigma }}_{ij}P_{j}^{\left(2\right)}P_{i}^{\left(2\right)}P_{j}^{\left(2\right)}+H({\hat{\bar{\sigma }}}^{\left(3\right)})P_{i}^{\left(3\right)}{\bar{\sigma }}_{ij}P_{j}^{\left(3\right)}P_{i}^{\left(3\right)}P_{j}^{\left(3\right)}$$

(41) $$\begin{array}{l} P^{+}=\sum_{k=1}^{3}H({\hat{\bar{\sigma }}}^{\left(k\right)})({P_{i}}^{\left(k\right)}{P_{j}}^{\left(k\right)}{P_{i}}^{\left(k\right)}{P_{j}}^{\left(k\right)})\\ P^{-}=I-P^{+} \end{array}$$

(42) $${{\bar{\sigma }}_{ij}}^{+}=P^{+}\bar{\sigma };\;{{\bar{\sigma }}_{ij}}^{-}=\bar{\sigma }-{{\bar{\sigma }}_{ij}}^{+}=P^{-}\bar{\sigma }$$

where the symmetric tensors $P^{+}$,$P^{-}$ are the positive and negative projection tensors of $\bar{\sigma }$, which are defined as:

(43) $$\begin{array}{l} P^{+}=\sum_{k=1}^{3}H({\hat{\bar{\sigma }}}^{\left(k\right)})({P_{i}}^{\left(k\right)}{P_{j}}^{\left(k\right)}{P_{i}}^{\left(k\right)}{P_{j}}^{\left(k\right)})\\ P^{-}=I-P^{+} \end{array}$$

where ${\hat{\bar{\sigma }}}^{\left(k\right)}$ are the principal stresses and H(·) is the Heaviside function.

Separate tensile and compressive damage components make it possible to distinguish the two loading-induced damage mechanisms. Under multiaxial or reverse-cyclic loading, however, both components may evolve simultaneously, and interaction between two damage surfaces can cause convergence difficulties [20]. To avoid this conflict, a single combined damage measure is formed by weighting the tensile and compressive damage variables as follows [25]:

(44) $$d=\frac{〚{\bar{\sigma }}_{ij}^{-}〛d^{-}+〚{\bar{\sigma }}_{ij}^{+}〛d^{+}}{〚{\bar{\sigma }}_{ij}〛}$$

where $〚{\bar{\sigma }}_{ij}〛={\bar{\sigma }}_{ij}{\bar{\sigma }}_{ij}$.

Based on the spectral decomposition of the effective stress tensor, the Helmholtz free energy can be correspondingly separated into tensile and compressive contributions. According to the thermodynamic definition of the damage energy release rate given in Equation (16), the tensile and compressive damage energy release rates are defined as the thermodynamic forces conjugate to the tensile and compressive damage variables $d^{+}$ and $d^{-}$, respectively:

(45) $$Y^{\pm }=-\frac{\partial \psi^{e}}{\partial d^{\pm }}$$

Thus, $Y^{+}$ and $Y^{-}$ represent the energy-driving forces associated with tensile and compressive damage evolution, respectively.

Substituting Equations (33) into Equation (45), gives:

(46) $$Y^{\pm }=-\frac{\partial \psi^{e}}{\partial d^{\pm }}=\frac{1}{2}\frac{〚{{\bar{\sigma }}_{ij}}^{\pm }〛}{〚{\bar{\sigma }}_{ij}〛}\left[\left({\bar{s}}_{ij}{\bar{D}}_{ijkl}{\bar{s}}_{kl})+\frac{1}{2}\beta (Y)(\frac{1}{9}{\left({\bar{\sigma }}_{mm}\right)}^{2}\delta_{ij}{\bar{D}}_{ijkl}\delta_{kl}\right)\right]$$

#### 3.2.3. Damage Formulation

$g^{\pm }$ is taken as a damage surface function of the corresponding damage energy release rate and damage variable [20]:

(47) $$g^{\pm }=Y^{\pm }-{Y_{0}}^{\pm }-Z^{\pm }$$

where $Z^{\pm }$ is a function of $d^{\pm }$ that controls the development of the damage surface.

Using the orthogonal flow rule, the evolution equation for the damage variable follows [19,26]:

(48) $${\dot{d}}^{\pm }={{\dot{\lambda }}_{d}}^{\pm }\frac{\partial g^{\pm }}{\partial Y^{\pm }}$$

Loading or unloading can be expressed through the Kuhn–Tucker relations:

(49) $${{\dot{\lambda }}_{d}}^{\pm }\geq 0,\;g^{\pm }\leq 0,\;{{\dot{\lambda }}_{d}}^{\pm }g^{\pm }=0$$

When $g^{\pm }\leq 0$, Equation (49) gives ${\dot{\lambda }}_{d}^{\pm }=0$, ${\dot{d}}^{\pm }=0$: the current damage criterion has not been violated yet, indicating unloading or neutral loading. During damage loading ${\dot{\lambda }}_{d}^{\pm }\geq 0$ Equation (49) gives $g^{\pm }=0$, and Equation (50) gives:

(50) $$g^{\pm }=Y^{\pm }-{Y_{0}}^{\pm }-Z^{\pm }=0$$

This paper defines [20]:

(51) $$Z^{\pm }=\frac{1}{a^{\pm }}{\left(\frac{d^{\pm }}{1-d^{\pm }}\right)}^{\frac{1}{b^{\pm }}}$$

(52) $$\mathrm{Thus}:\;d^{\pm }=G^{\pm }(Y^{\pm })=1-\frac{1}{1+{\left(a^{\pm }Z^{\pm }\right)}^{b^{\pm }}}=1-\frac{1}{1+{\left(a^{\pm }[Y^{\pm }-Y_{0}^{\pm }]\right)}^{b^{\pm }}}$$

When $d^{\pm }$ is evaluated, direct use of Equation (47) to form the combined scalar damage d does not reproduce the observed response under combined tension and compression [17]. Therefore, the combined scalar damage relation of Lee and Fenves [24] is modified here:

(53) $$d=1-(1-sd^{+})(1-d^{-})$$

where $s=\left\|{\sigma_{ij}}^{+}\right\|/\left\|\sigma_{ij}\right\|$.

#### 3.2.4. The Helmholtz Free Energy of This Paper

In the Tao–Phillips formulation, the damage reduction factor makes the damage energy release rate nonlinear and requires a local iteration within each strain increment. This increases the constitutive solution complexity and does not remove all discrepancies under multiaxial loading.

**Figure 2:**
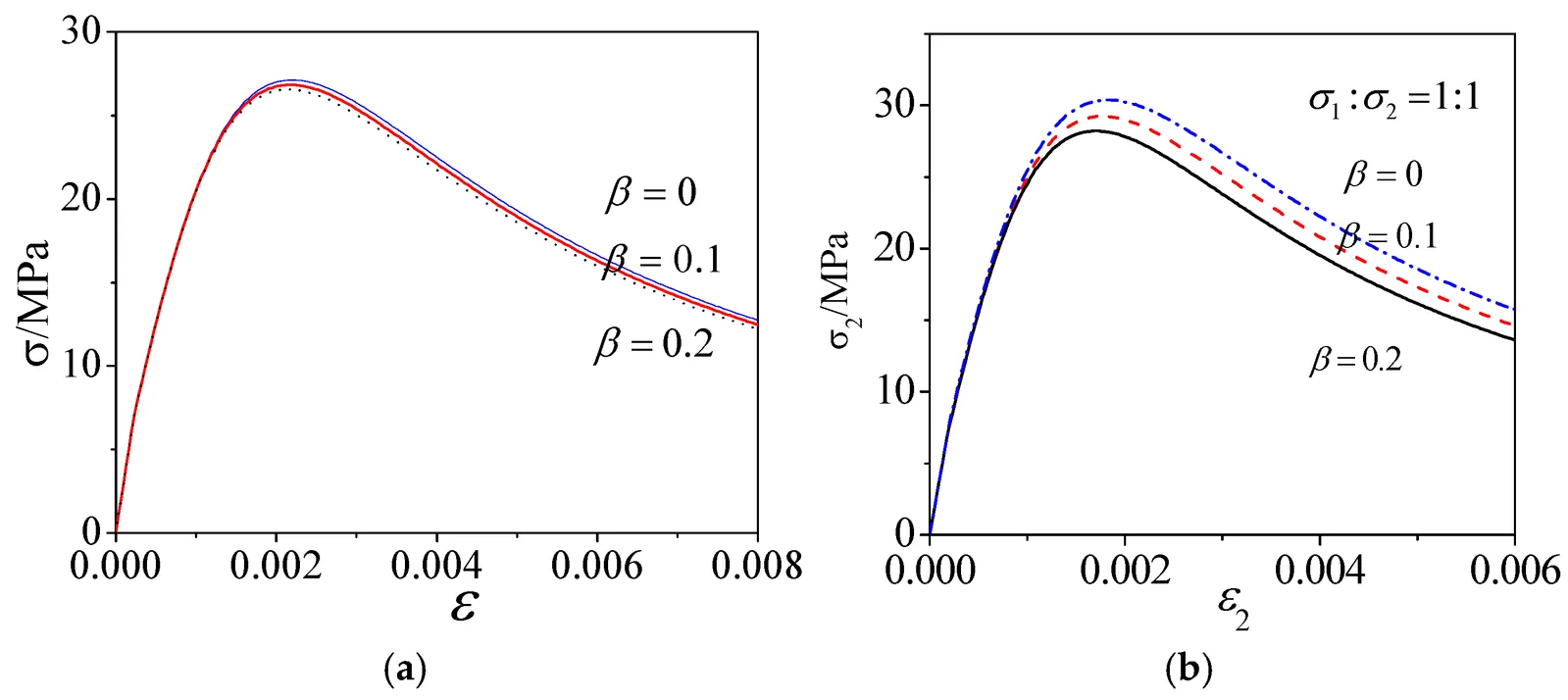
Uniaxial and biaxial compression. (**a**) 1D compressive; (**b**) 2D compressive.

**Figure 3:**
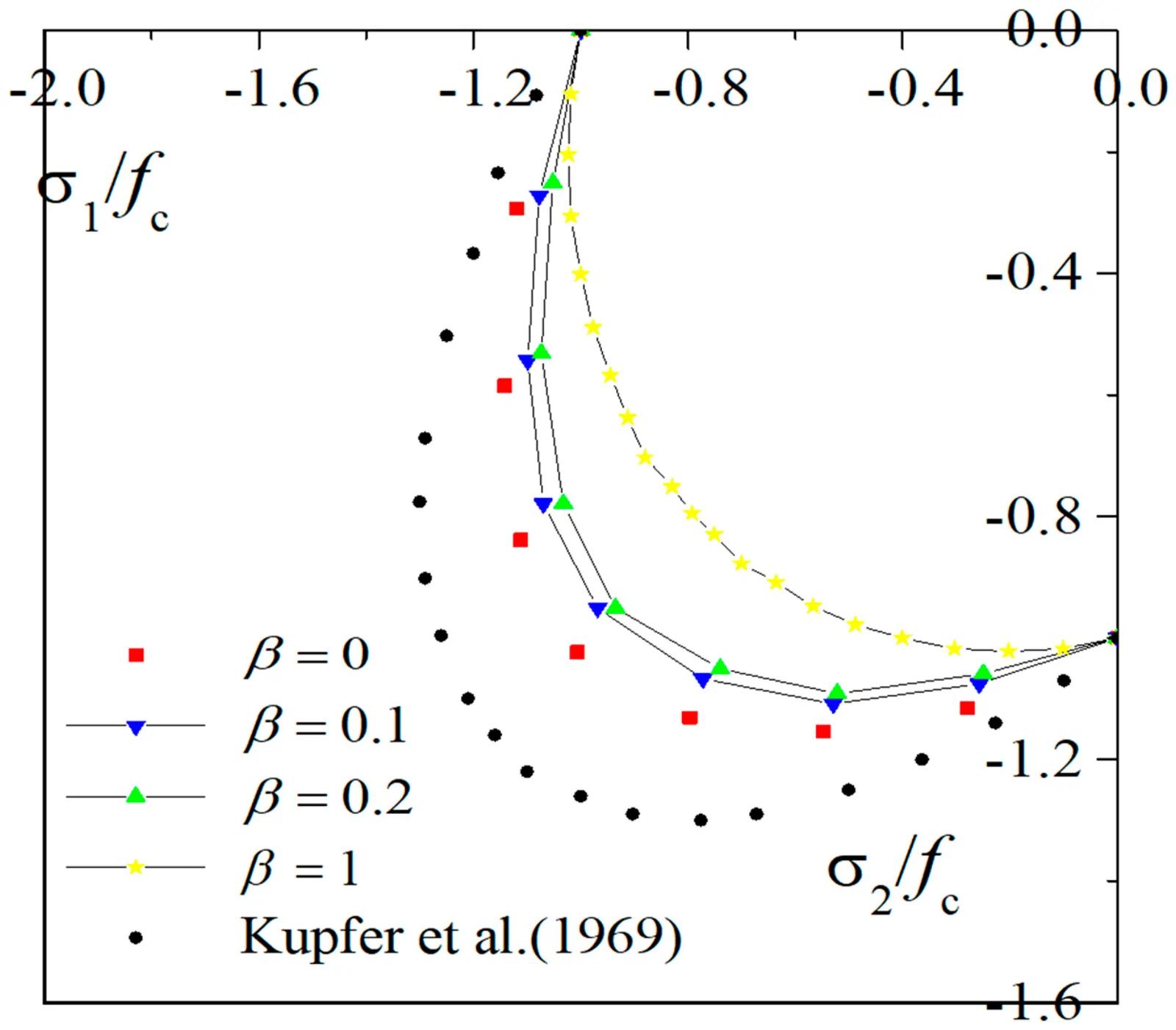
Biaxial strength envelope [28].

**Figure 4:**
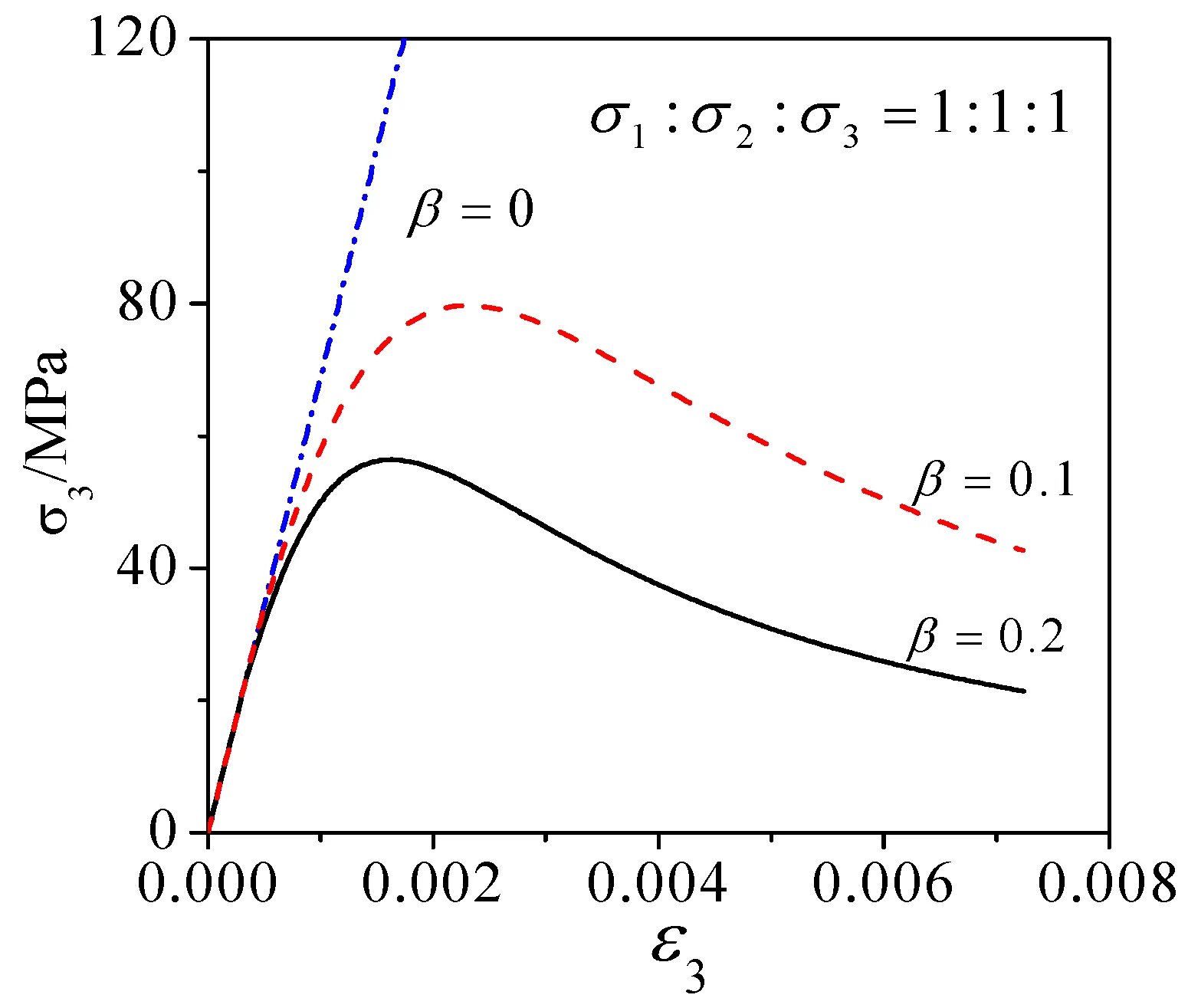
Triaxial compression.

The effect of β on the stress–strain curve of concrete under 1D, 2D, and 3D compressive loading is plotted in Figure 2, Figure 3 and Figure 4; material properties are E=31000MPa, v=0.2, $f_{c}=27.6\mathrm{MPa}$, $a^{-}=28{\mathrm{MPa}}^{-1}$, $b^{-}=1.0$, $\beta_{p}=0.1+0.45{\hat{\sigma }}_{1}/{\hat{\sigma }}_{2}$, $Y_{0}^{-}=0.031f_{c}^{2}/E$.

From Figure 2, one can see that the change in β has little effect on the stress–strain curve under 1D compression. However, the influence of β becomes more pronounced under biaxial compression. From Figure 3, one can see that as β decreases, the simulation result becomes increasingly close to the test results.

**Figure 5:**
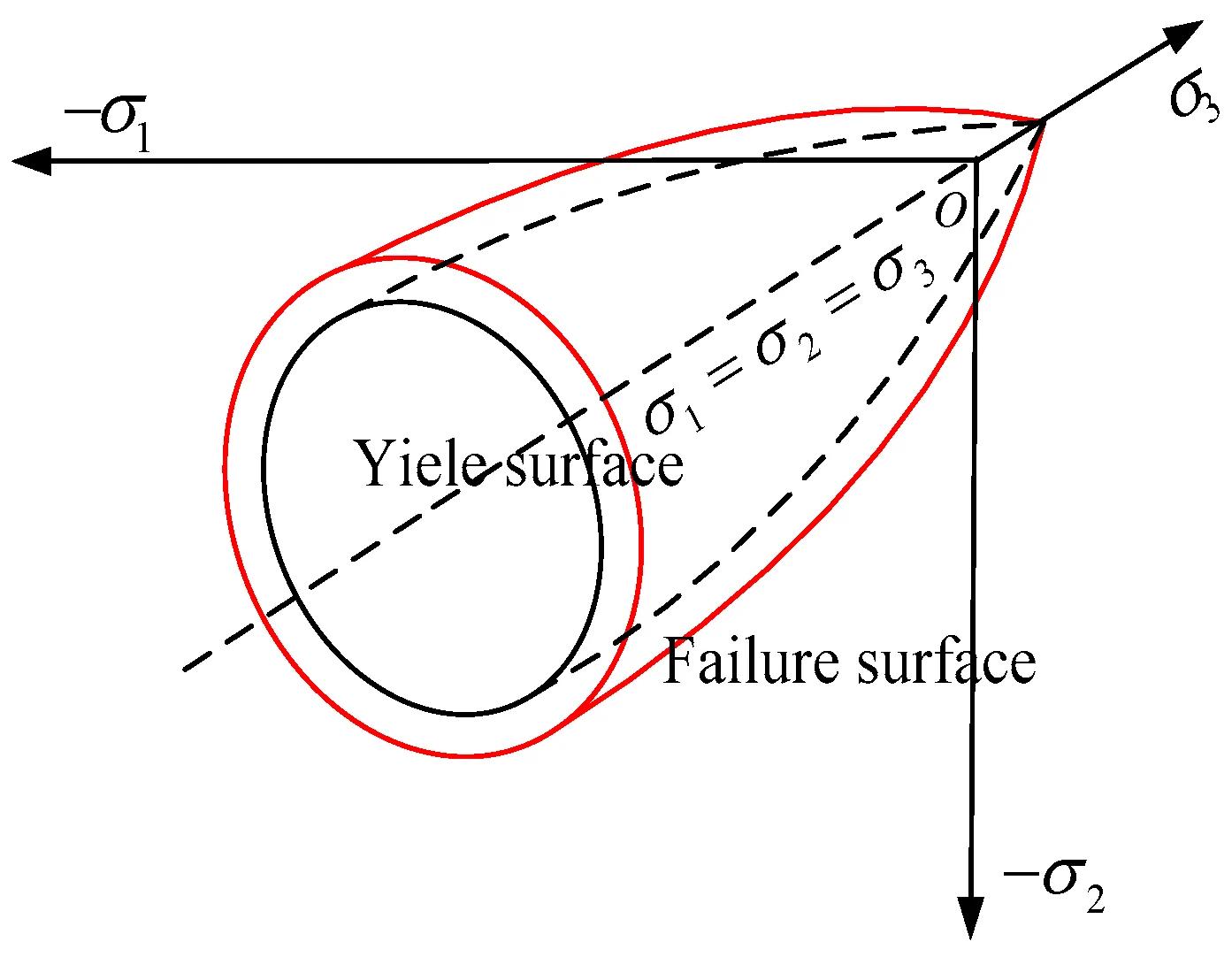
The yield surface and fracture surface of concrete.

From Figure 4, one can see that under hydrostatic pressure, if β=0 the ultimate strength of concrete is infinite; however, if β=0.1 and β=0.2 the calculated ultimate strengths are about 50MPa and 80 MPa. One can see from the typical failure surface and yield surface of concrete [27] (see Figure 5) that they do not show a trend of closing under hydrostatic pressure. Therefore, the numerical results presented above support the adoption of β=0 for the loading conditions considered in the present study.

The adoption of β=0 also eliminates the local iteration associated with the original β-dependent formulation and simplifies the evaluation of the damage energy release rate. It should be emphasized that setting β=0 does not mean that hydrostatic pressure has no influence on concrete damage. Rather, it means that the hydrostatic component of the Helmholtz free energy is not considered a direct contribution to the damage-driving force.

When β=0, Equation (46) can be simplified as follows:

(54) $$Y^{\pm }=-\frac{\partial \psi^{e}}{\partial d^{\pm }}=\frac{1}{2}\frac{〚{{\bar{\sigma }}_{ij}}^{\pm }〛}{〚{\bar{\sigma }}_{ij}〛}({\bar{s}}_{ij}{\bar{D}}_{ijkl}{\bar{s}}_{kl})$$

Although the hydrostatic energy component is excluded from the direct damage-driving force, the overall hydrostatic stress state can still influence damage evolution indirectly. In particular, the same or similar deviatoric stress state may produce different damage responses under different confinement levels. A higher confining pressure can reduce the damage-producing effect of the deviatoric stress component.

For example, under a uniaxial stress state $\left(\sigma_{0},0,0\right)$ and a biaxial stress state $\left(\sigma_{0},\sigma_{0},0\right)$, the deviatoric stresses have different components, but a damage criterion based only on the deviatoric contribution may not sufficiently distinguish their different multiaxial responses. Based on the above analysis, this paper introduces a stress-state-dependent coefficient γ into Equation (54):

(55) $$Y^{\pm }=\frac{1}{2}\frac{〚{{\bar{\sigma }}_{ij}}^{\pm }〛}{〚{\bar{\sigma }}_{ij}〛}\gamma ({\bar{s}}_{ij}{\bar{D}}_{ijkl}{\bar{s}}_{kl})$$

where γ is a dimensionless coefficient used to modify the damage-producing contribution of the deviatoric stress component according to the current stress state. Therefore, β and γ play different roles in the present formulation: β controls the direct contribution of the hydrostatic free energy component, whereas γ accounts for the indirect influence of the multiaxial stress state on the deviatoric damage-driving force.

To provide a simple correction for the stress states considered in the present study, γ is expressed as:

(56) $$\gamma =1-0.265(1-H({\hat{\sigma }}_{2}))\sqrt{\left|{\hat{\sigma }}_{2}/{\hat{\sigma }}_{3}\right|}$$

Here, ${\hat{\sigma }}_{2}$ and ${\hat{\sigma }}_{3}$ denote the second and third principal stresses, respectively. The function $H\left(.\right)$ is the Heaviside function.

**Figure 6:**
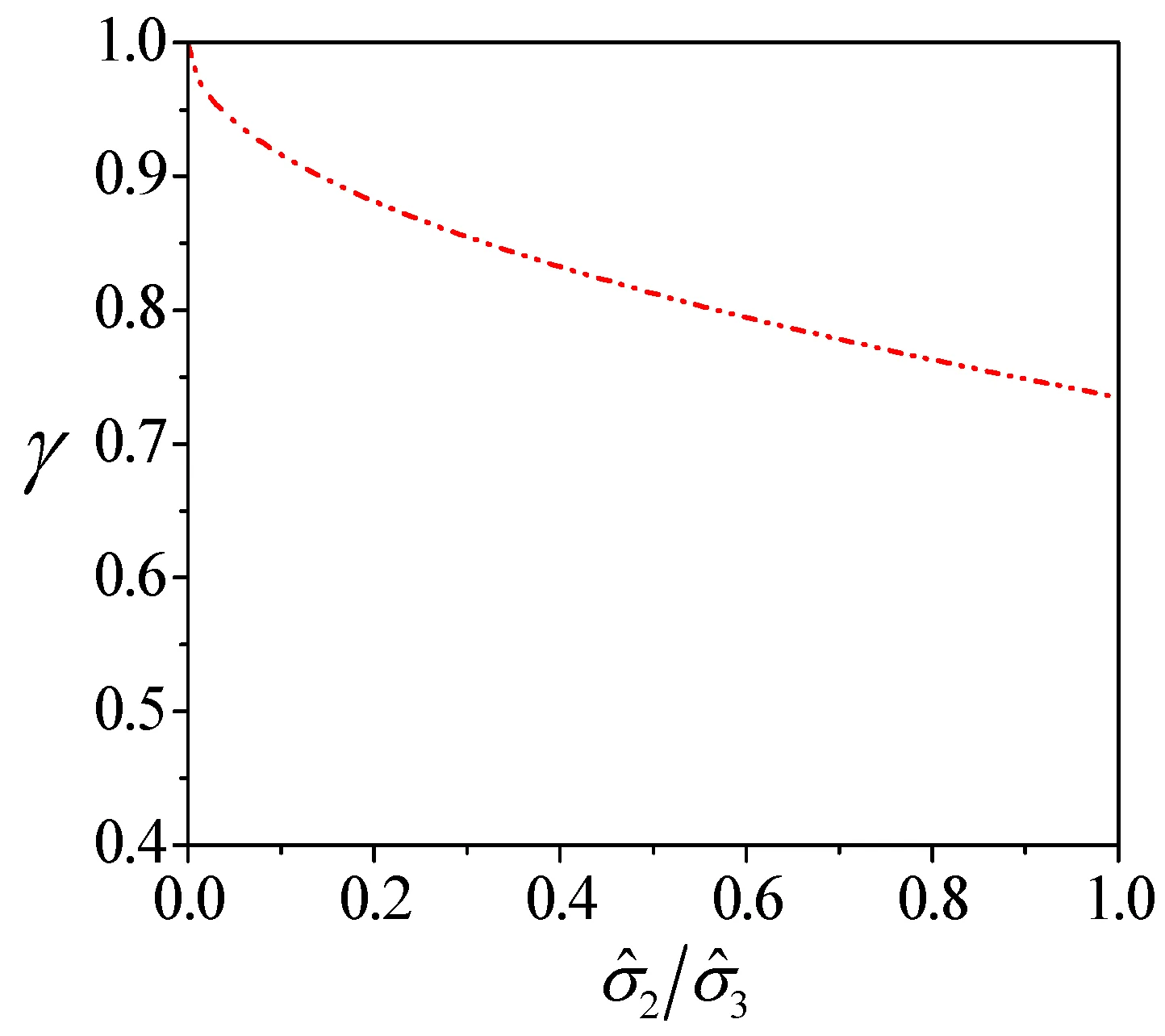
The relationship between γ and ${\hat{\sigma }}_{2}/{\hat{\sigma }}_{3}$.

The numerical coefficient 0.265 in Equation (56) is an empirical value adopted in the present study. It was determined by inverse fitting through a series of trial calculations against the experimental data used in the present validation, so as to obtain agreement between the simulated and measured responses. Therefore, this coefficient is not regarded as a universal material constant and should be recalibrated when the model is applied to a different concrete material or dataset. The relationship between γ and ${\hat{\sigma }}_{2}/{\hat{\sigma }}_{3}$ is show in Figure 6.

The numerical results of the model for the biaxial compressive strength envelope are compared to test results reported by Kupfer et al. [28]. The comparison is used to evaluate the improvement in the biaxial response obtained by introducing the stress-state correction coefficient γ.

For 3D loading, the introduction of γ can improve the calculation accuracy; however, the present form of γ has not yet been comprehensively calibrated for general three-dimensional stress states, particularly under high confinement. Therefore, further validation using systematic triaxial experimental data is required before extending the present expression to more general 3D loading conditions.

The present formulation does not require an explicit definition of a conventional plastic strength/failure surface. Concrete deterioration is governed by the modified Helmholtz free energy and the corresponding damage evolution, while plastic strain is evaluated in the effective stress space. Accordingly, a prescribed Rankine tension meridian, circular deviatoric section, or explicit Lode-angle-dependent failure surface is not directly introduced in the present formulation. A systematic sensitivity analysis of the stress-state coefficient γ, together with further calibration under general three-dimensional and triaxial stress states, will be carried out in future work.

For practical application of the proposed model, the main material parameters can be identified from conventional uniaxial and multiaxial concrete tests. The tensile and compressive damage parameters are determined independently from the corresponding tensile and compressive responses, whereas $\beta_{p}$ mainly controls irreversible deformation and γ accounts for the influence of the multiaxial stress state. Table 1 summarizes the physical meaning and the recommended identification procedure for the principal model parameters. The parameter values adopted in the validation examples are determined according to the corresponding material properties and experimental responses rather than being treated as universal constants.

The tensile and compressive damage parameters should therefore be calibrated separately because the proposed model distinguishes tensile and compressive damage evolution. The parameter $\beta_{p}$ is mainly related to irreversible deformation and is more appropriately identified from cyclic tests containing unloading and residual strain information. In contrast, γ cannot be identified from a uniaxial test alone because its role is to distinguish the damage response under different multiaxial stress states.

For a different concrete material, the parameter ranges listed in Table 1 should be regarded as initial estimates. The tensile damage parameters should be recalibrated using the corresponding uniaxial tensile stress–strain response, the compressive damage parameters using the uniaxial compressive response, $\beta_{p}$ using unloading–reloading and residual-strain data, and γ using multiaxial experimental data.

### 3.3. The Consistent Elastic-Plastic Damage Tangent Operator

For implicit finite-element analysis, equilibrium is solved with a Newton–Raphson scheme; the corresponding material tangent is therefore required. The consistent tangent operator for the present model is derived below.

Form $\sigma_{ij}=(1-d){\bar{\sigma }}_{ij}$ we can obtain:

(57) $$\frac{\partial \sigma_{ij}}{\partial \varepsilon_{ij}}=(1-d)\frac{\partial {\bar{\sigma }}_{ij}}{\partial \varepsilon_{ij}}-\frac{\partial d}{\partial \varepsilon_{ij}}{\bar{\sigma }}_{ij}$$

(58) $$\frac{\partial d}{\partial \varepsilon_{ij}}=\frac{\partial d}{\partial \varepsilon_{ij}^{e}}\frac{\partial \varepsilon_{ij}^{e}}{\partial \varepsilon_{ij}};\;\frac{\partial \varepsilon_{ij}^{e}}{\partial \varepsilon_{ij}}=E_{mnpq}^{-1}\frac{\partial {\bar{\sigma }}_{ij}}{\partial \varepsilon_{ij}}$$

Substituting Equation (58) into Equation (57), gives:

(59) $$\frac{\partial \sigma_{ij}}{\partial \varepsilon_{ij}}=(\delta_{ij}\delta_{kl}-d\delta_{ij}\delta_{kl}-\frac{\partial d}{\partial \varepsilon_{ij}^{e}}E_{mnpq}^{-1}{\bar{\sigma }}_{ij})\frac{\partial {\bar{\sigma }}_{ij}}{\partial \varepsilon_{ij}}$$

(60) $$\frac{\partial {\bar{\sigma }}_{ij}}{\partial \varepsilon_{ij}}=C_{0}-\beta_{p}EH({\dot{d}}^{-})\langle I_{{\bar{\sigma }}_{ij}}{\dot{\varepsilon }}_{ij}\rangle \langle I_{{\bar{\sigma }}_{ij}}⊗I_{{\bar{\sigma }}_{ij}}\rangle$$

(61) $$\frac{\partial d}{\partial \varepsilon_{ij}^{e}}=s(1-d^{-})\frac{\partial d^{+}}{\partial \varepsilon_{ij}^{e}}+(1-sd^{+})\frac{\partial d^{-}}{\partial \varepsilon_{ij}^{e}}$$

(62) $$\frac{\partial d^{\pm }}{\partial \varepsilon_{ij}^{e}}=\frac{\partial d^{\pm }}{\partial Y^{\pm }}\frac{\partial Y^{\pm }}{\partial \varepsilon_{ij}^{e}}$$

(63) $$\frac{\partial d^{\pm }}{\partial Y^{\pm }}=\frac{a^{\pm }b^{\pm }{\left(a^{\pm }[Y^{\pm }-Y_{0}^{\pm }]\right)}^{(b^{\pm })-1}}{{\left(1+{\left(a^{\pm }[Y^{\pm }-Y_{0}^{\pm }]\right)}^{b^{\pm }}\right)}^{2}}$$

(64) $$\left\{\begin{array}{c} \frac{\partial Y^{+}}{\partial \varepsilon_{ij}^{e}}=\frac{\left\|{\sigma_{ij}}^{+}\right\|}{\left\|\sigma_{ij}\right\|}({\bar{E}}_{ijkl}{\varepsilon_{kl}}^{e}-\frac{1}{9}(({\varepsilon_{mn}}^{e})\delta_{ij}{\bar{E}}_{ijkl}\delta_{kl}))\\ \frac{\partial Y^{-}}{\partial \varepsilon_{ij}^{e}}=\frac{\left\|{\sigma_{ij}}^{-}\right\|}{\left\|\sigma_{ij}\right\|}\gamma ({\bar{E}}_{ijkl}{\varepsilon_{kl}}^{e}-\frac{1}{9}(({\varepsilon_{mn}}^{e})\delta_{ij}{\bar{E}}_{ijkl}\delta_{kl})) \end{array}\right.$$

By substituting Equations (60)–(64) into Equation (59), we can calculate the tangent stiffness modulus.

### 3.4. Computational Algorithm

**Figure 7:**
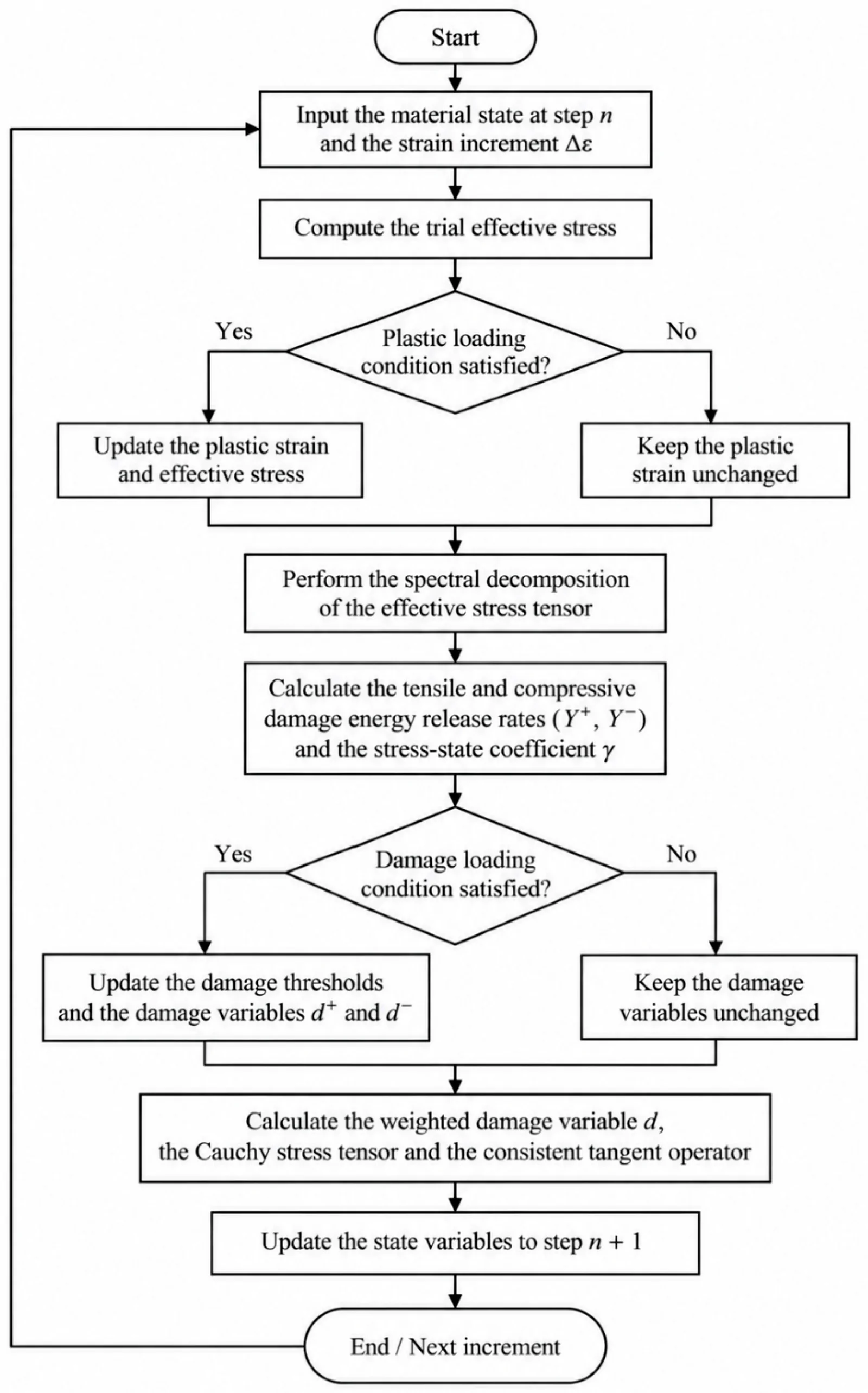
Flowchart of the constitutive update algorithm for the proposed elastic–plastic damage model.

The implementation of the proposed model is relatively straightforward because the plastic strain and damage variables can be evaluated explicitly. The constitutive response is updated incrementally. The material state at the beginning of a loading increment is denoted by n, and the updated state at the end of the increment is denoted by *n* + 1. The overall constitutive update procedure is summarized in Figure 7.

The above procedure is repeated for each loading increment until the prescribed loading history is completed.

## 4. Applications

### 4.1. The Biaxial Strength Envelope of Concrete

**Figure 8:**
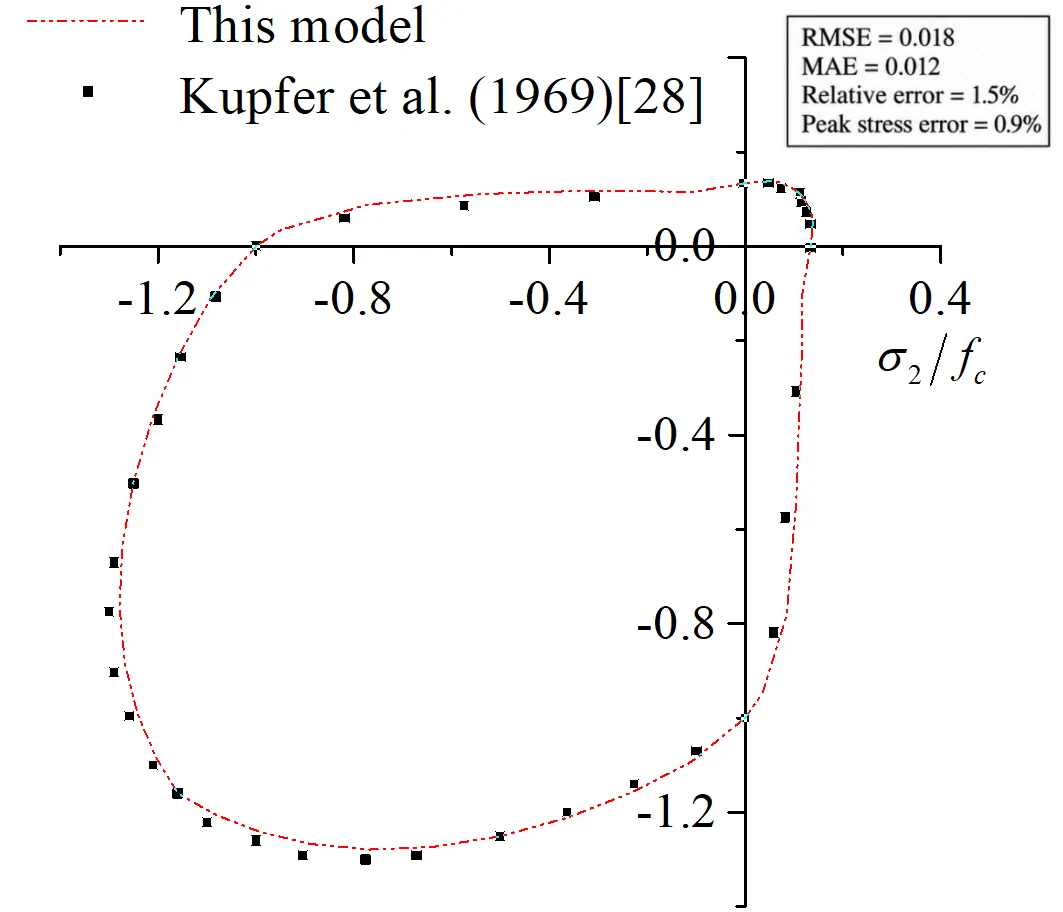
Biaxial strength envelope of concrete [28].

Figure 8 compares the numerical predictions of the proposed model with the experimental results reported by Kupfer et al. [28]. The model reproduces the overall shape and variation in the biaxial strength envelope under pure tension, pure compression, and combined tension–compression stress states. mage model for concrete matesimulation are E=31000MPa, v=0.2, $f_{c}=27.6\mathrm{MPa}$, $f_{t}=3.5\mathrm{MPa}$, $a^{-}=28$, $a^{+}=7000{\mathrm{MPa}}^{-1}$, $b^{-}=1$, $b^{+}=1.1$, $\beta_{p}=0.1+0.45{\hat{\sigma }}_{1}/{\hat{\sigma }}_{2}$, $Y_{0}^{+}=0.5f_{t}^{2}/E$, $Y_{0}^{-}=0.031f_{c}^{2}/E$.

### 4.2. Monotonic Uniaxial, Biaxial Compressive Test

**Figure 9:**
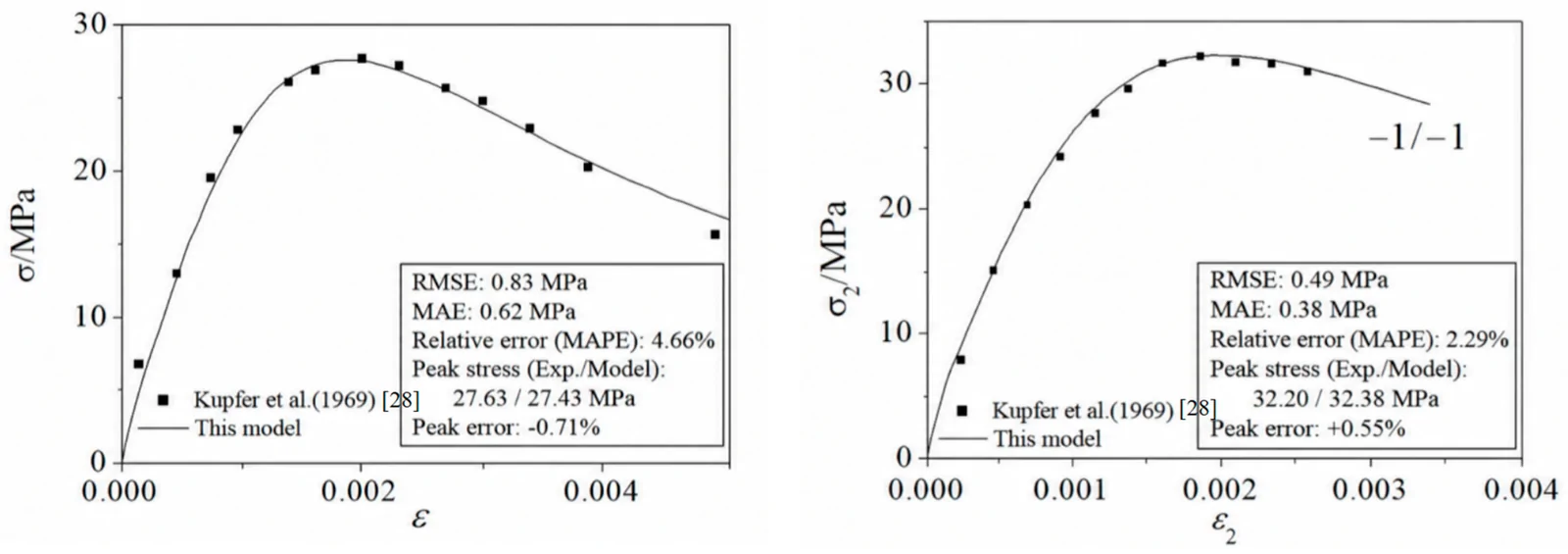
1D and 2D compressive tests [28]. (**left**) 1D compressive test; (**right**) 2D compressive test.

Figure 9 compares the numerical responses predicted by the proposed model with the experimental results reported by Karsan and Jirsa [29] and Kupfer et al. [28] under monotonic uniaxial and biaxial compression. The numerical results reproduce the main characteristics of the experimental stress–strain responses, including the initial stiffness, peak response, and post-peak softening behavior. The material properties used in the simulations are E=26000MPa, v=0.2, $f_{c}=32\mathrm{MPa}$, $a^{-}=17.2$, $b^{-}=1.4$, $\beta_{p}=0.53$, $Y_{0}^{-}=0.031f_{c}^{2}/E$; E=31800MPa, v=0.2, $f_{t}=3.4\mathrm{MPa}$, $a^{+}=7000$, $b^{+}=1.1$, $Y_{0}^{+}=0.5f_{t}^{2}/E$.

### 4.3. Uniaxial Cyclic Compression and Tension Tests

**Figure 10:**
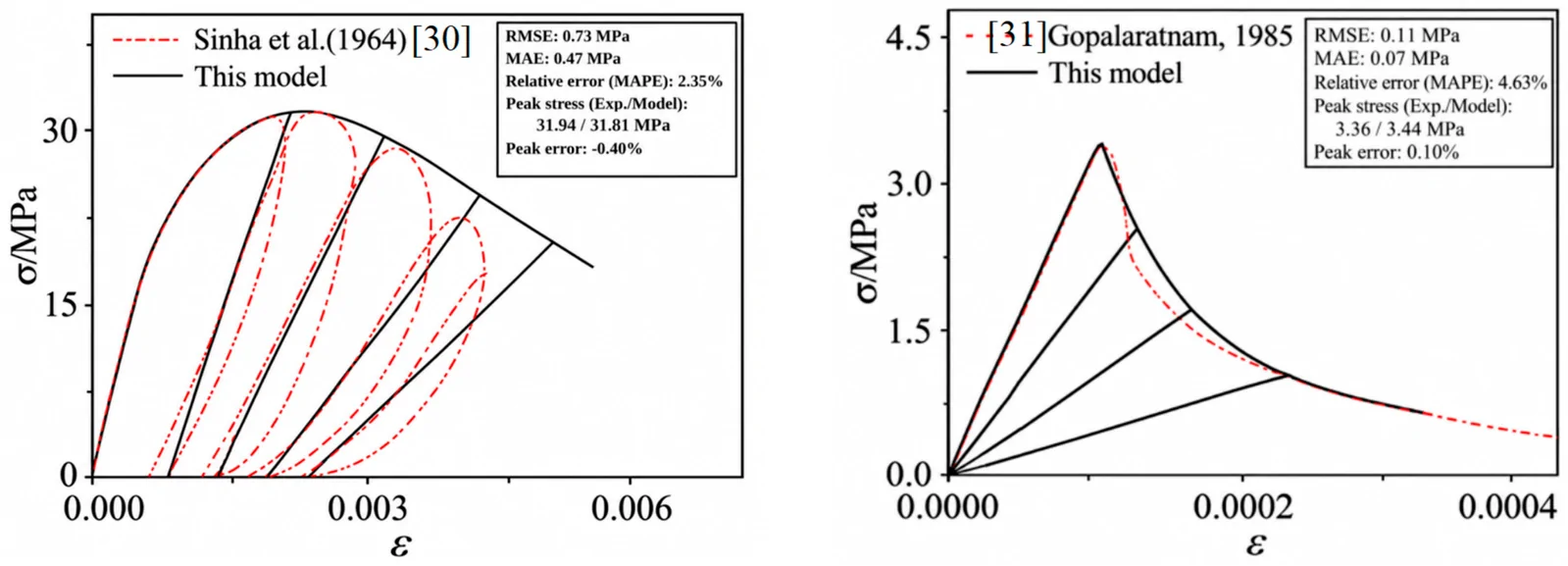
Uniaxial cyclic compression and tension tests [30,31].

In this section, the performance of the proposed model under uniaxial cyclic compression and tension is investigated. Figure 10 compares the numerical predictions with the experimental results reported by Sinha et al. [30] and Gopalaratnam and Shah [31]. The comparison is used to evaluate the model response under cyclic loading, including stiffness degradation, unloading–reloading behavior, and residual deformation. The material properties used here are E=26000MPa, $f_{c}=31.9MPa$, $a^{-}=17.2$, $b^{-}=1.4$, $\beta_{p}=0.53$, $f_{t}=3.4MPa$; E=31800MPa, $a^{+}=7000$, $b^{+}=1.1$, $Y_{0}^{-}=0.031f_{c}^{2}/E$, $Y_{0}^{+}=0.5f_{t}^{2}/E$ according to Equation (29).

It should be noted that the present formulation does not reproduce irreversible strain under pure tensile loading. This is a limitation of the current model rather than an assumption intended to represent the actual tensile behavior of concrete. In the present formulation, irreversible deformation is mainly associated with compressive damage and plastic evolution, whereas tensile nonlinearity is primarily represented through stiffness degradation and tensile damage. Therefore, the model is more suitable for applications in which compressive plastic deformation is dominant. Additional tensile plasticity would be required for problems in which permanent deformation under repeated or predominant tensile loading plays an important role.

**Figure 11:**
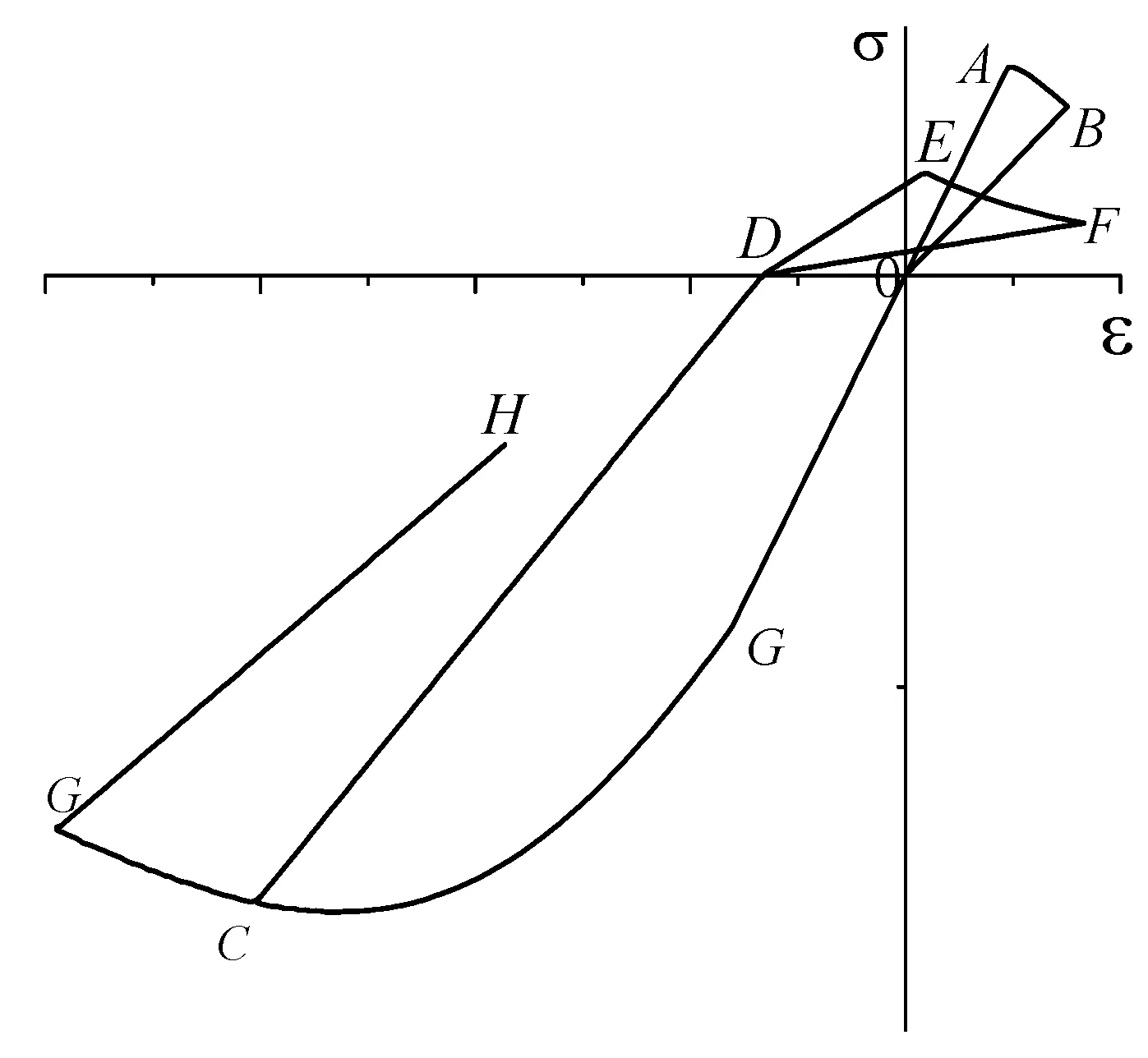
Cyclic behavior during a uniaxial test.

Figure 11 presents the numerical response for successive tension–compression cycles: the first tension loading–unloading cycle (OABO), the first compression loading–unloading cycle (OGCD), the second tension loading–unloading cycle (DEFD), and the second compression loading–unloading cycle (DCGH).

## 5. The Numerical Simulation of the Koyna Concrete Dam

### 5.1. Damping

Experimental vibration studies of concrete dams commonly report damping ratios of about 2–5% [24]. In the present model, damping is represented by a stiffness-proportional term. The mass-proportional Rayleigh component is omitted because no physical basis is assigned to it in the adopted formulation. For the undamaged material, the damping stress is written as:

(65) $${\bar{\sigma }}_{vis}=\beta_{k}{\bar{E}}_{ijkl}{\dot{\varepsilon }}_{ij}$$

Here, β_k denotes the stiffness-proportional damping coefficient, and ε̇_ij denotes the strain-rate tensor.

The viscous damping stress contribution is evaluated as:

(66) $$\sigma_{vis}=(1-d){\bar{\sigma }}_{vis}=\beta_{k}(1-d){\bar{E}}_{ijkl}{\dot{\varepsilon }}_{ij}$$

For the present plastic damage formulation:

(67) $$\sigma_{ij}=(1-d){\bar{\sigma }}_{ij},\;{\bar{\sigma }}_{ij}={\bar{E}}_{ijkl}\varepsilon_{ij}^{e}={\bar{E}}_{ijkl}(\varepsilon_{ij}-\varepsilon_{ij}^{p})$$

The total stress then becomes:

(68) $$\sigma_{tot}=\sigma_{ij}+\sigma_{vis}=(1-d)({\bar{\sigma }}_{ij}+{\bar{\sigma }}_{vis})$$

After inclusion of stiffness-proportional damping, the elastic–plastic damage tangent operator is:

(69) $$\frac{\partial \sigma_{tot}}{\partial \varepsilon_{ij}}=\frac{\partial \sigma_{ij}}{\partial \varepsilon_{ij}}+\frac{d\sigma_{vis}}{d\varepsilon_{ij}}$$

(70) $$\frac{d\sigma_{vis}}{d\varepsilon_{ij}}=\beta_{k}(1-d){\bar{E}}_{ijkl}\frac{1}{dt}$$

### 5.2. Analysis of Damage to Koyna Dam

The proposed model is applied to the Koyna concrete dam. The material parameters are $\rho_{0}=2643kg/m^{3}$, E=31027MPa, $\nu_{0}=0.2$, $\beta_{p}=0.5$, the tensile strength is estimated to be $f_{t}=2.9MPa$, and the compressive strength is $f_{c}=24.1MPa$. The stiffness damping parameter is $\beta_{k}=0.0033$ to provide 3% damping at the fundamental vibration period. The full reservoir is represented by added masses at the upstream face nodal points. The finite element model uses 760 four-node plane-strain quadrilateral isoparametric elements.

**Figure 12:**
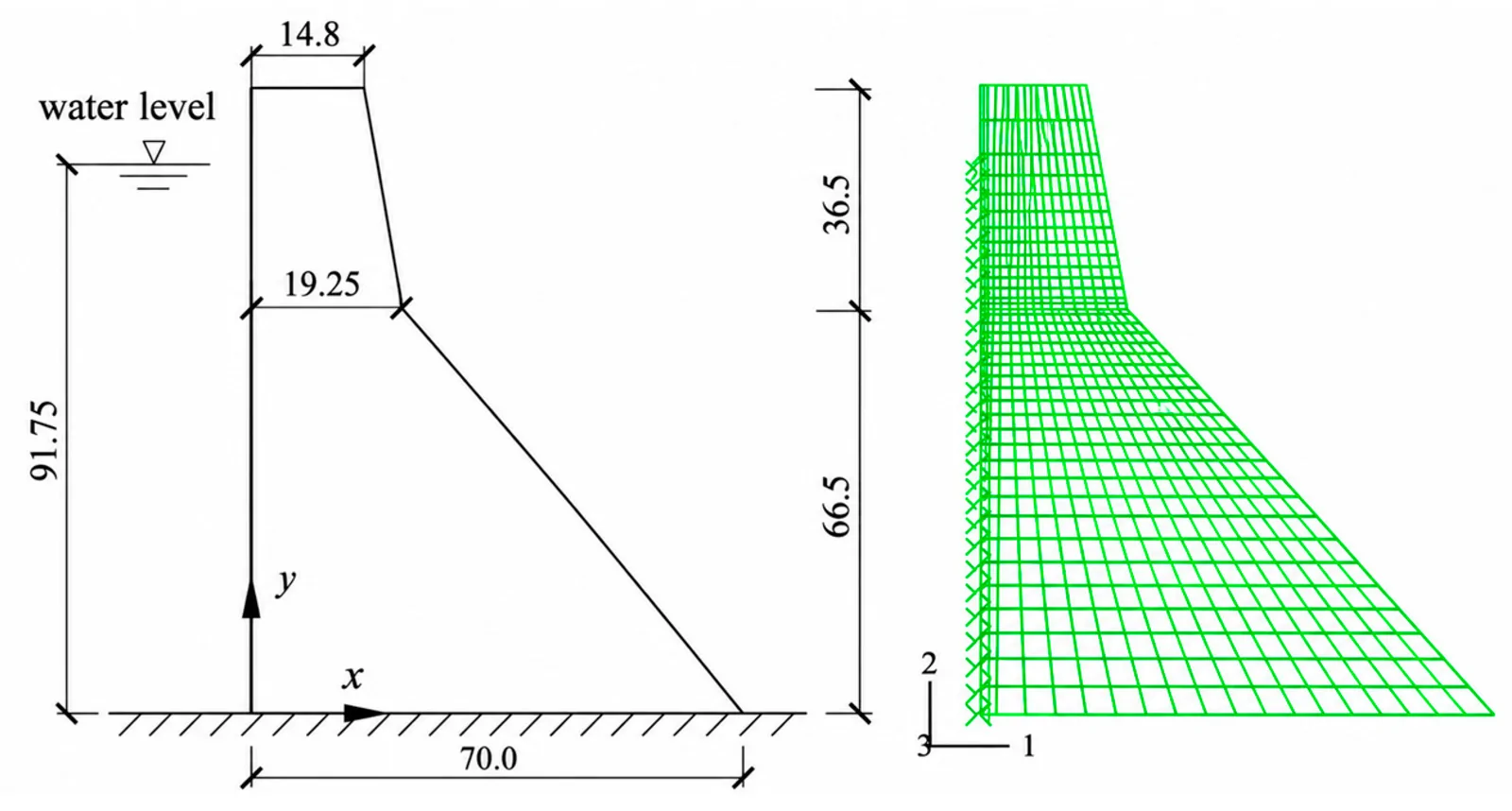
Geometry and finite element discretization of the Koyna concrete gravity dam.

The selected non-overflow monolith of the Koyna dam has a maximum height of 103 m, a crest width of 14.8 m, and a base width of 70.2 m. The dam base is fully fixed. The applied loads consist of dam self-weight, upstream hydrostatic pressure, and the horizontal and vertical acceleration time histories recorded during the Koyna earthquake. The hydrodynamic effect of reservoir water is represented using the Westergaard added-mass method. The geometry and finite element discretization of the Koyna concrete gravity dam are shown in Figure 12.

From Table 2, we can see that the results of this model match well with Chopra and Chakrabarti [32] and Lee and Fenves [24].

**Figure 13:**
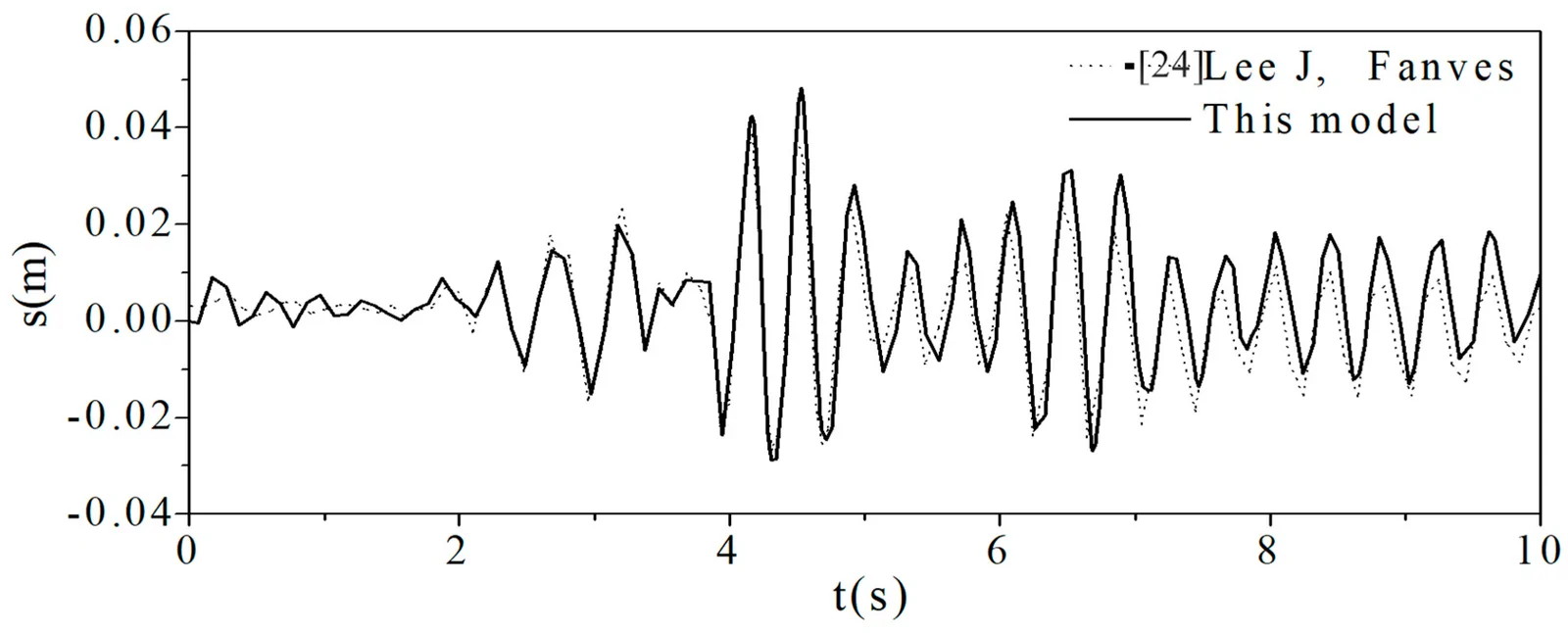
Results of the rate-independent model [24].

Figure 13 compares the present numerical response with that reported by Lee and Fenves [24]; the two responses show close agreement.

**Figure 14:**
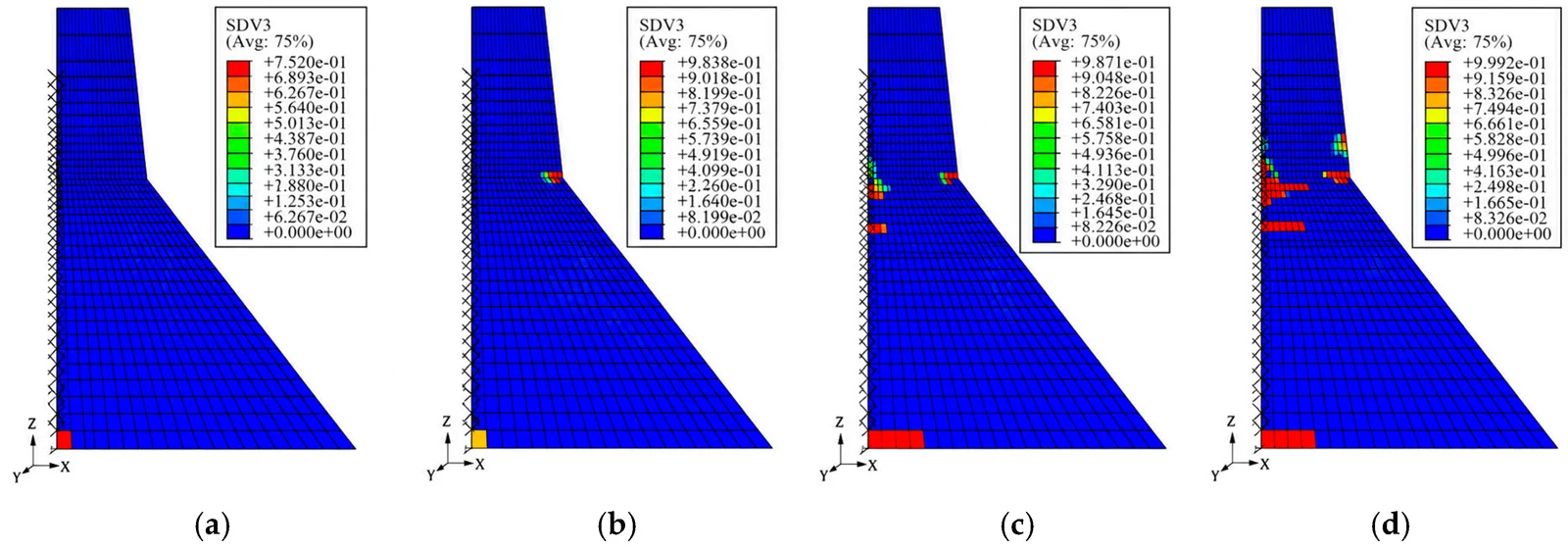
Development of tensile damage. (**a**) 2.735(s); (**b**) 3.977(s); (**c**) 4.164(s); (**d**) 10(s).

Figure 14 illustrates the evolution of tensile damage. At 2.735 s, cracking initiates at the upstream side of the base and propagates into the dam. By 3.997 s, a horizontal crack develops at the downstream face near the stress concentration at the change in downstream slope. At 4.164 s, another horizontal crack initiates on the upstream face and rapidly advances into the dam.

## 6. Conclusions

The main conclusions of this study are summarized as follows:

(1) The proposed formulation combines effective stress-space plasticity with elastic–plastic damage mechanics for concrete. Plastic deformation and damage evolution are decoupled to simplify the constitutive formulation. Two scalar damage variables and a plastic strain tensor are adopted as internal variables. Within the framework of continuum damage mechanics, the elastic damage energy release rate is decomposed into hydrostatic and deviatoric components. The hydrostatic free energy component is excluded from the direct damage-driving force, while the stress-state-dependent coefficient γ is introduced to account for the influence of the multiaxial stress state on the deviatoric damage-driving contribution. A weighted damage variable is further introduced to describe the interaction between tensile and compressive damage.

(2) The model is validated against experimental results under uniaxial, biaxial, and cyclic loading conditions. The numerical results reproduce the main characteristics of the experimental responses, including strength evolution, stiffness degradation, and post-peak behavior. The application to the Koyna concrete dam further demonstrates the applicability of the proposed model to nonlinear structural analysis. The first three natural circular frequencies obtained from the present model are 18.86, 49.98, and 68.16 rad/s, which are close to the corresponding results reported by Chopra and Chakrabarti [32] and Lee and Fenves [24].

(3) The present formulation still has several limitations. In particular, it does not reproduce irreversible strain under pure tensile loading, because irreversible deformation is mainly associated with compressive damage and plastic evolution in the current model. In addition, the present form of the stress-state coefficient γ has not yet been comprehensively calibrated for general three-dimensional stress states, particularly under high confinement. Future work should therefore focus on the incorporation of tensile plasticity, further calibration of γ using triaxial experimental data, and validation under more general cyclic and three-dimensional loading conditions. Future work will also include a systematic sensitivity study of γ, direct benchmarking against standard CDP formulations using unified datasets and parameter identification procedures, and quantitative evaluation of computational cost and convergence behavior.

## Figures and Tables

**Table 1:** Identification of the main parameters of the proposed model.

Parameter	Physical Meaning	Source	Value
$a^{+}$	Controls the evolution rate of tensile damage	Calibrated from the post-peak branch of a uniaxial tensile stress–strain curve	6000~8000
$b^{+}$	Controls the shape of tensile damage evolution	Calibrated together with $a^{+}$ using the tensile softening response	1.0~1.2
$Y_{0}^{+}$	Initial threshold for tensile damage	Determined from the stress/strain level corresponding to the onset of tensile damage	$0.5f_{t}^{2}/E$
$a^{-}$	Controls the evolution rate of compressive damage	Calibrated from the nonlinear and post-peak portions of the compressive stress–strain curve	15~20
$b^{-}$	Controls the shape of compressive damage evolution	Calibrated together with $a^{-}$ using the compressive response	1.3~1.5
$Y_{0}^{-}$	Initial threshold for compressive damage	Determined from the stress/strain level corresponding to the onset of compressive damage	$0.031f_{c}^{2}/E$
$\beta_{p}$	Controls the magnitude of irreversible plastic strain evolution	Identified primarily from residual strain and unloading–reloading behavior	0.5~0.55
γ	Stress-state correction of the deviatoric damage-driving contribution	Calibrated using multiaxial strength and stress-state-dependent response	Equation (56)

**Table 2:** Comparison of the first three natural circular frequencies of the Koyna concrete dam.

Vibration Model	Nature Frequency (rad/s)		
	This Model	Chopra A.K	Lee, J
1	18.86	19.27	18.55
2	49.98	51.5	49.14
3	68.16	67.56	67.02

## Data Availability

The original contributions presented in this study are included in the article. Further inquiries can be directed to the corresponding author.
